# Chewing Discomfort According to Dental Prosthesis Type in 12,802 Adults: A Cross-Sectional Study

**DOI:** 10.3390/ijerph18010071

**Published:** 2020-12-24

**Authors:** Jae-Hyun Lee, Da Hye Kim, Yong-Gyu Park, Su Young Lee

**Affiliations:** 1 Department of Prosthodontics and Dental Research Institute, School of Dentistry, Seoul National University, 101 Daehak-ro, Jongro-gu, Seoul 03080, Korea; jaehyun.lee@snu.ac.kr; 2 Department of Biostatistics, College of Medicine, The Catholic University of Korea, 222 Banpo-daero, Seocho-gu, Seoul 06591, Korea; dahaene1228@naver.com (D.H.K.); ygpark@catholic.ac.kr (Y.-G.P.); 3 Department of Prosthodontics, Seoul St. Mary’s Dental Hospital, College of Medicine, The Catholic University of Korea, 222 Banpo-daero, Seocho-gu, Seoul 06591, Korea

**Keywords:** big data, chewing discomfort, dental prosthesis, dentures, mastication

## Abstract

This study examined the prevalence of self-perceived chewing discomfort depending on the type of dental prosthesis used in South Korean adults. The subjects were 12,802 people over 20 years of age who participated in a health interview and dental examination. Chewing discomfort was examined using a self-assessed report with a structured questionnaire. Using multivariable logistic regression analysis, adjusted odds ratios were evaluated along with their 95% confidence intervals (α = 0.05). After adjusting for covariates, including age, gender, smoking, drinking, hypertension, diabetes, body mass index, education, income, and toothbrushing frequency, the odds ratios (95% confidence intervals) for chewing discomfort in groups without a dental prosthesis, with fixed dental prostheses, with removable partial dentures, and with removable complete dentures were 1 (reference), 1.363 (1.213–1.532), 2.275 (1.879–2.753), and 2.483 (1.929–3.197), respectively. The association between the prevalence of chewing discomfort and the type of dental prosthesis used was statistically significant even after adjusting for various confounders (*p* < 0.0001). The type of dental prosthesis was related to chewing discomfort among South Korean adults.

## 1. Introduction

Masticatory function has often been measured by crushing tests, usually using several types of sieve systems [1]. However, since the measurement of masticatory efficiency by laboratory methods has shown a weak interrelation between self-assessment and experimental measurements, patient-based assessments have been suggested as more suitable for the estimation of chewing ability [2]. Many concepts of masticatory function have been identified [2,3,4,5,6]. Among these, chewing ability is a personal and subjective assessment of masticatory function, while masticatory performance or efficiency measures the ability to reduce the size of food particles. Some studies have suggested that masticatory efficiency and chewing ability are related to the number and location of remaining teeth [3,4,5,6]. The health of dentition and chewing ability has far-reaching significance, and important associations have been identified between chewing ability and cognitive disorders and brain functions [7,8,9,10], as well as between chewing ability and quality of life [11,12,13,14,15]. Moreover, an impaired oral condition has been associated with increased mortality [16,17,18]. Chewing, therefore, plays a critical role in the daily lives of all individuals. It is critical to analyze the distribution of chewing ability through a population-based household survey to understand its dysfunctions in the adult and elderly populations. However, little is known about the prevalence of chewing discomfort according to the type of dental prosthesis in the adult population. Thus, this study evaluated the effect of the type of dental prosthesis on chewing discomfort. This study aimed to identify the prevalence of self-reported chewing discomfort depending on the type and status of dental prosthesis used in an adult South Korean population to assist in treatment planning and the provision of prosthetic management.

## 2. Materials and Methods

### 2.1. Survey Overview

This study was conducted using data from the sixth Korean National Health and Nutrition Examination Survey (KNHANES VI), which was performed from 2013 to 2015. The KNHANES was first carried out as a nationwide survey in 1998 by the Division of Chronic Disease Surveillance of the Korea Centers of Disease Control and Prevention.

### 2.2. Participants

A total of 22,948 KNHANES VI participants had adequate data available for inclusion in this study. The KNHANES VI was approved by the Institutional Review Board (approval numbers, 2013-07CON-03-4C and 2013-12EXP-03-5C) for Human Subjects of the Korea Centers for Disease Control and Prevention (now the Korea Disease Control and Prevention Agency). Written informed *p* for trend was obtained from all participants. The inclusion criteria of the current study were as follows: (i) Aged 20 years or older, (ii) the presence of oral examination data, and (iii) no missing data on the variables considered in the analyses. After applying the inclusion criteria, the definitive sample size for the study was 12,802. The study protocol adhered to the ethical principles for medical research involving human subjects, as defined by the Declaration of Helsinki.

### 2.3. Sociodemographic Status

The sociodemographic variables examined were age, gender, household income, education level, and residence. Household income was divided into 4 quartiles for different age/gender groups with regard to the mean monthly equalized household income. Education level was divided into 4 groups. Residence location was divided into rural or urban areas.

### 2.4. General Health Status

Trained examiners collected the general health status data from survey participants. The body mass index (BMI) was calculated as weight in kilograms (kg) divided by the square of the height in square meters (m^2^). The BMI cut-off point was 25 kg/m^2^ for obesity according to the World Health Organization recommendations for Asian populations [19]. The health behavior variables included smoking and drinking. The stress levels from all participants were assessed as none, mild, moderate, or severe. Participants with moderate or severe stress were categorized into the stress group. Diabetes was identified as a fasting blood sugar concentration of ≥126 mg/dL or currently taking antidiabetic medication. Hypertension was defined as a systolic blood pressure ≥140 mmHg, diastolic blood pressure ≥90 mmHg, or currently taking systemic antihypertensive drugs.

### 2.5. Oral Health Status

Specially trained dentists performed oral health evaluations following the guidelines of oral health surveys for dental examinations using mouth mirrors and dental probes under artificial light. The number of natural teeth and prosthetic status were recorded. We categorized the cohort into 4 groups according to the type of dental prosthesis as follows: Having only natural teeth without any dental prosthesis, having tooth-supported or implant-supported fixed dental prostheses without any removable denture, having removable partial dentures but not removable complete dentures, and having at least 1 removable complete denture [20]. Dental pain was categorized into throbbing toothache, dull or aching tooth, or pain in the teeth when eating a cold or hot beverage or food during the last year. Prosthesis need was evaluated by the dentist at the time of examination. The frequency of tooth brushing was also surveyed based on the reported number of tooth brushing events per day [21].

### 2.6. Evaluation of Chewing Discomfort

A trained interviewer examined the participants using a questionnaire developed for this survey. The self-assessed presence of chewing discomfort was determined from a dental health-related behavior examination. The questions were structured as follows: “Do you have difficulties with chewing food because of intraoral problems, including teeth, dentures, or gums? (If you use dentures, please describe your experience with respect to wearing dentures.)” The possible answers to “chewing problems” were written based on a 5-step point scale to indicate that the discomfort was very much (1), quite a lot (2), slight (3), very little (4), or not at all (5). This investigation divided chewing discomfort into not problematic (very little, not at all) and problematic (very much, quite a lot, slightly).

### 2.7. Statistical Analysis

Chi-square tests for categorical variables and *t*-tests for continuous variables satisfying normal assumption were used to assess the general characteristics of the participants and the distributions of dental prostheses. Multivariate logistic regression analysis was used to analyze the odds ratios (ORs) and 95% confidence intervals (CIs) for the prevalence of chewing discomfort according to the type of dental prosthesis. We utilized SAS version 9.2 (SAS Institute Inc., Cary, NC, USA) to calculate approximations of the entire adult Korean population and to account for the complicated sample model. All statistical procedures were two-tailed, and *p* < 0.05 was considered statistically significant.

## 3. Results

**Figure 1:**
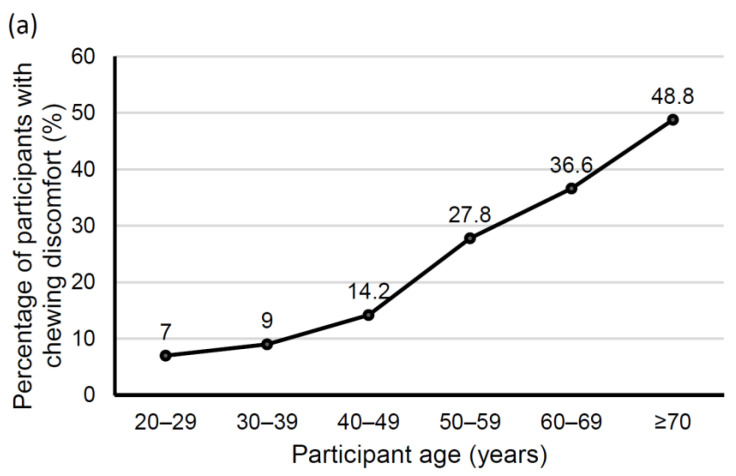

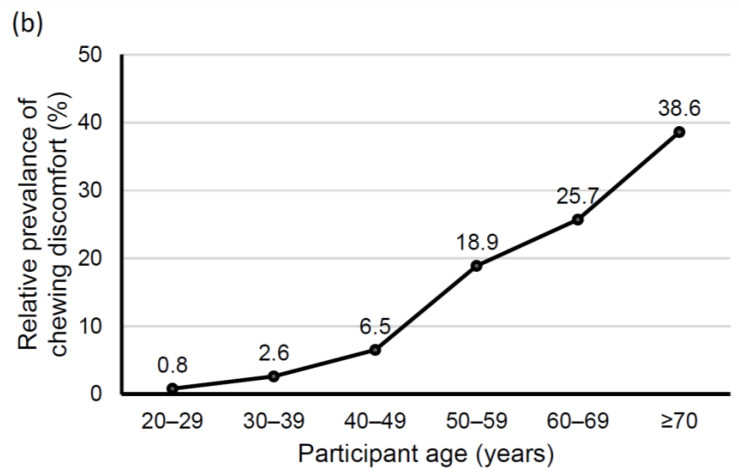
Chewing discomfort according to age classes. (**a**) Percentage of participants with chewing discomfort (*p* for trend < 0.0001). (**b**) Relative prevalence of chewing discomfort for dental prosthesis users (*p* for trend < 0.0001).

Table 1 presents the general features of adults with or without chewing discomfort, of whom 23.25% reported chewing discomfort. The participants with chewing discomfort were older, and higher percentages of people with lower income and education level had chewing discomfort compared to those with higher income and education. Chewing discomfort was more common in rural residents than in urban residents. Participants with higher BMI, obesity, current smoking, drinking, stress, diabetes, and hypertension showed higher percentages of chewing discomfort (all *p* < 0.05). Dental pain, denture need, and fewer remaining teeth were more prevalent in participants with chewing discomfort than in participants without chewing discomfort (*p* < 0.0001). Among those with chewing discomfort, the percentage of participants with tooth brushing frequencies of twice or less was higher than that of those who brushed three or more times per day (*p* < 0.0001). The percentage of participants with chewing discomfort increased with age classes (*p* for trend < 0.001). In Figure 1a, chewing discomfort increased markedly in the 50s (27.8%), and nearly half (48.8%) of the participants in their 70s and over showed chewing discomfort. Figure 1b shows the relative prevalence of chewing discomfort among participants using fixed and/or removable dental prostheses. The relative prevalence of chewing discomfort for dental prosthesis users was also found to increase significantly with age classes (*p* for trend < 0.001).

Table 2 shows the distributions of participants depending on the type of dental prosthesis used. Our findings showed that age, gender, income, education, residence, BMI, obesity, current smoking, alcohol consumption, stress, diabetes, hypertension, dental pain, dental clinic visit during the previous year, number of remaining teeth, and the daily frequency of tooth brushing differed significantly according to the distributions with the type of dental prosthesis (*p* < 0.05).

**Figure 2:**
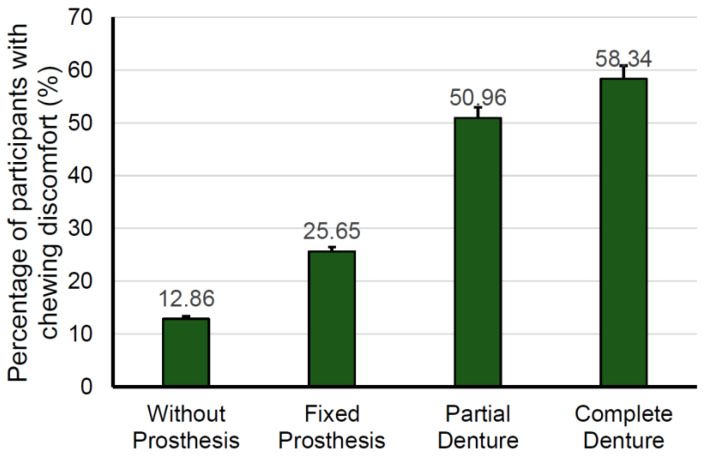
Percentage of participants with chewing discomfort according to dental prosthesis type (*p* for trend < 0.0001).

Figure 2 shows the proportions of chewing discomfort according to the types of dental prostheses. The prevalence of chewing discomfort in participants without dental prosthesis was 12.86%. The prevalence of chewing discomfort for participants with complete dentures (58.34%) was the highest compared to that for participants with partial dentures (50.96%) and fixed prosthesis (25.65%) (*p* for trend < 0.0001).

Table 3 shows the multivariate-adjusted odds ratios (ORs) for having chewing discomfort depending on each dental prosthesis. According to the results presented in Table 1 and Table 2, Models 1–4 were sequentially adjusted for potential confounders showing a significant relationship with chewing discomfort or dental prosthesis use. The models were gradually adjusted for variables related to demographic characteristics (Model 1), general health status (Model 2), socioeconomic status (Model 3), and oral health behavior (Model 4). Regarding the reference group (without prosthesis), the ORs for chewing discomfort increased from fixed prosthesis to removable denture after adjusting for all covariates (OR = 1.363 and OR = 2.275 in fixed prosthesis and partial denture, respectively, and OR = 2.483 in complete denture; 95% confidence interval (*p* for trend < 0.0001)).

## 4. Discussion

The current study was performed to assess the prevalence of self-perceived chewing discomfort according to the type of dental prosthesis used in South Korean adults based on an analysis of a well-organized, large national population-based survey. Self-assessed chewing ability is considered an appropriate means for evaluating mastication, and based on its practicality demonstrated elsewhere, self-evaluation was selected as a method of assessing chewing ability in this study. Although several studies have assessed chewing ability in human subjects [3,4,5,6], few studies have investigated the prevalence of chewing discomfort according to the dental prosthesis type in all age groups of the adult population [22]. Therefore, we investigated the chewing ability according to the type of dental prosthesis used in Korean adults.

Chewing discomfort increased significantly with increase in age. In particular, almost half of the participants aged 70 and over showed chewing discomfort (48.8%). The outcomes of the current study correspond to those of previous reports that chewing ability tended to decrease with age [23,24]. In 2018, 14.3% of the South Korean population was aged 65 years or older, a proportion expected to continue to grow as the life expectancy of Koreans increases. Thus, the number of individuals with chewing discomfort will increase. This implies the necessity for the development and application of new dental care services for elderly people with chewing discomfort.

In addition, the financial status and the education level significantly influenced both chewing discomfort (Table 1) and the type of dental prosthesis used (Table 2). According to epidemiologic studies, educational level and economic status are closely related and have been shown to have an important effect on the prevalence and type of dental prosthesis [25,26,27]. In this study, higher percentages of people with lower income and education level had chewing discomfort compared to those with higher income and education. This may not mean that more expensive prostheses are of better quality. However, the cost of fixed dental prosthesis increases with the number of missing teeth requiring rehabilitation, whereas removable prostheses can replace many teeth at a lower cost.

Residents of rural regions also showed a higher prevalence of removable denture use and chewing discomfort. This difference may be associated with issues in the accessibility of dental clinics. Dental care is more frequently limited in rural areas. Individual preferences might also play an important role. People living in rural regions may be less demanding in their oral health than urban residents and more likely to accept removable prostheses over a fixed dental prosthesis [28].

Adults with complete dentures complained of chewing discomfort 2.483-times more frequently than those without prosthesis and with natural teeth (*p* < 0.0001) (Table 3). These findings contribute to a better understanding of the relationship between chewing ability and dental status in adults. Removable denture wearers usually prefer softer foods because the maximum masticatory force strength of the person wearing complete dentures is lower than that of dentate individuals. Previous studies reported that complete denture wearers exhibited a maximum biting force that was one-seventh to one-quarter of that of an average person with intact dentition [29,30]. However, these differences could be caused by differences in the ages of the participants. Thus, this study tried to identify the independent relationship by adjusting for various potential confounders, including age.

There are inherent differences in the propagation of the actual force between complete denture wearers and people with natural teeth. Persons with natural teeth tend to generate force through the bolus, whereas most of the chewing force is distributed outside of the bolus directly through the denture base onto the supporting tissues in complete denture wearers. Therefore, complete denture wearers show reduced consumption of food that is more difficult to masticate, such as fresh fruits, vegetables, and meats, due to their diminished chewing ability. These changes in food consumption habits induce nutritional deficiencies in vitamins, minerals, fiber, and proteins and lead them to consume more calories from sources higher in carbohydrates, fats, and cholesterol [31,32,33]. These food consumption changes may be related to obesity or underweight among complete denture users. Thus, people with chewing discomfort require proper treatment.

In this study, the participants who used removable dental prostheses reported greater masticatory discomfort than those who used fixed prostheses. If a specially trained prosthodontist carefully restores a removable dental prosthesis, it may yield good results comparable to a fixed dental prosthesis. However, since this study analyzed national representative big data rather than data from individual university hospitals, this result may have been observed due to uncontrolled fabrication and loss of maintenance visits for some removable dental prostheses. Removable dental prostheses are usually less stable than fixed dental prostheses but have the advantage of being easier to clean and easy to examine for pathologic lesions beneath the prosthesis [34]. Therefore, it might be beneficial for clinicians and patients to plan for prosthetic treatment with consideration of patients’ functional needs and comfort.

This study has some limitations. This was a cross-sectional study from which we cannot confirm causality. In addition, since data on chewing difficulties were obtained using a self-assessed questionnaire, masticatory capabilities may be underestimated when a subjective assessment method is used. Nevertheless, the present study still has many strengths. The data analyzed in this study were acquired from the large representative population of the Korea National Health Survey. The adjusted models were used to analyze the prevalence of chewing discomfort and dental prosthesis. In addition, most of the previous studies performed on this topic have concentrated on the association between chewing ability and the number of missing teeth [3,4,5,6]. However, most of the areas with missing teeth do not remain edentulous. The missing teeth may usually be rehabilitated with fixed and/or removable dental prostheses. Thus, the present study evaluated chewing discomfort according to the dental prosthesis used.

## 5. Conclusions

Despite the limitations of the cross-sectional design, the results of this study showed that the type of dental prosthesis was significantly associated with perceived chewing discomfort. Complete denture wearers showed a 2.483-fold increase in the risk of chewing discomfort, which remained significant after adjusting for various confounders. When examining complete denture users in dental clinics, it may be beneficial to carefully evaluate their masticatory discomfort. Additional longitudinal studies are needed to better understand this association.

## Figures and Tables

**Table 1:** General characteristics of participants with and without chewing discomfort.

Variable		Chewing Discomfort		*p*-Value
		No	Yes	
Unweighted (n)	12802	9825	2977	
Total (%)	100	76.75	23.25	
Age (years)		42.89 ± 0.23	55.41 ± 0.38	<0.0001
Gender	Male	50.05 (0.5)	49.81 (1.02)	0.8413
	Female	49.95 (0.5)	50.19 (1.02)	
Income	Lowest quartile	10.86 (0.51)	26.21 (1.06)	<0.0001
	Lower middle quartile	24.01 (0.79)	27.44 (1.08)	
	Upper middle quartile	31.39 (0.85)	24.41 (1.02)	
	Highest quartile	33.74 (1.03)	21.94 (1.14)	
Education	Elementary: ≤6 y	10.43 (0.41)	34.61 (1.2)	<0.0001
	Middle: 7–9 y	7.47 (0.31)	14.79 (0.83)	
	High: 10–12 y	40.68 (0.7)	31.11 (1.19)	
	University: ≥ 13 y	41.42 (0.79)	19.49 (1.04)	
Place of residence	Rural	15.49 (1.47)	21.37 (1.92)	<0.0001
Body mass index (BMI, kg/m^2^)		23.67 ± 0.04	24.04 ± 0.08	<0.0001
Obesity	BMI ≥ 25	31.37 (0.54)	35.81 (1.08)	<0.0001
Smoking	current smoker	21.28 (0.55)	26.5 (1.09)	<0.0001
Drinking	heavy drinker	16.5 (0.48)	18.81 (0.9)	0.0169
Stress	Yes	24.67 (0.55)	31.57 (1)	<0.0001
Diabetes	Yes	7.21 (0.29)	16.68 (0.82)	<0.0001
Hypertension	Yes	20.72 (0.49)	37.11 (1.12)	<0.0001
Dental pain	Yes	33.55 (0.75)	57.57 (1.18)	<0.0001
Prosthesis need	Yes	4.12 (0.23)	20.97 (0.88)	<0.0001
Dental clinic visit (last year)	Yes	30.44 (0.63)	28.95 (1.06)	0.1945
Remaining teeth (n)		25.95 ± 0.05	21.25 ± 0.18	<0.0001
Tooth brushing frequency	≤1	8.89 (0.34)	16.9 (0.88)	<0.0001
	2	35.68 (0.54)	42.16 (1.12)	
	≥3	55.43 (0.59)	40.94 (1.14)	

Values are presented as mean ± SE or % (SE).

**Table 2:** Distributions of participants according to the dental prosthesis used.

		Without Prosthesis	Fixed Prosthesis	Partial Denture	Complete Denture	*p*-Value
Unweighted (n)	12802	7441	3812	914	635	
Total (%)	100	58.12	29.78	7.14	4.96	
Age (years)		39.36 ± 0.21	53.27 ± 0.3	66.05 ± 0.46	69.34 ± 0.55	<0.0001
Gender	Male	50.59 (0.58)	48.91 (0.84)	44.68 (1.83)	54.81 (2.14)	0.002
	Female	49.41 (0.58)	51.09 (0.84)	55.32 (1.83)	45.19 (2.14)	
Income	Lowest quartile	9.42 (0.53)	15.86 (0.76)	38.91 (1.91)	48.3 (2.6)	<0.0001
	Lower middle quartile	24.55 (0.87)	23.87 (0.93)	28.73 (1.9)	27.7 (2.27)	
	Upper middle quartile	32.26 (0.91)	28.42 (1.06)	19.63 (1.57)	14.39 (1.8)	
	Highest quartile	33.78 (1.08)	31.85 (1.23)	12.73 (1.4)	9.61 (1.59)	
Education	Elementary: ≤6 y	7.17 (0.35)	21.79 (0.88)	53.35 (2.07)	63.28 (2.46)	<0.0001
	Middle: 7–9 y	6.3 (0.33)	13.27 (0.63)	16.24 (1.47)	14.74 (1.85)	
	High:10–12 y	42.25 (0.76)	36.22 (0.98)	22.06 (1.94)	16.53 (1.8)	
	University: ≥13 y	44.29 (0.86)	28.73 (1.02)	8.34 (1.17)	5.45 (1.16)	
Place of residence	Rural	14.04 (1.43)	19.15 (1.73)	27.89 (2.7)	31.13 (3.39)	<0.0001
Body mass index (BMI, kg/m^2^)		23.58 ± 0.05	24.04 ± 0.06	24.35 ± 0.13	23.78 ± 0.17	<0.0001
Obesity	BMI ≥ 25	30.5 (0.6)	35 (0.93)	40.04 (1.99)	33.25 (2.38)	<0.0001
Smoking	Current smoker	23.36 (0.63)	20.02 (0.78)	18.93 (1.72)	24.58 (2.15)	0.0006
Drinking	Heavy drinker	17.29 (0.56)	17.15 (0.75)	13.06 (1.36)	14.58 (1.69)	0.0447
Stress	Yes	27.51 (0.65)	24.29 (0.87)	17.91 (1.48)	22.63 (1.98)	<0.0001
Diabetes	Yes	5.8 (0.28)	12.42 (0.65)	22.45 (1.71)	26.66 (2.2)	<0.0001
Hypertension	Yes	16.48 (0.54)	33.36 (0.87)	53.64 (1.98)	51.67 (2.58)	<0.0001
Dental pain	Yes	36.66 (0.83)	43.47 (0.97)	42.9 (2.12)	22.14 (2.17)	<0.0001
Dental clinic visit (last year)	Yes	28.85 (0.7)	37.49 (1.01)	21.3 (1.75)	10.94 (1.43)	<0.0001
Remaining teeth (n)		27.29 ± 0.02	23.96 ± 0.07	15.32 ± 0.22	3.7 ± 0.19	<0.0001
Tooth brushing frequency	≤1	8.84 (0.4)	9.66 (0.61)	19.99 (1.7)	34.3 (2.32)	<0.0001
	2	36.03 (0.64)	38.09 (0.93)	41.27 (1.81)	39.68 (2.38)	
	≥3	55.13 (0.67)	52.25 (1)	38.74 (2.04)	26.02 (2.01)	

Values are presented as mean ± SE or % (SE).

**Table 3:** Odds ratios of chewing discomfort in the participants with respect to the dental prosthesis type (95% confidence interval (CI)).

	Model 1	Model 2	Model 3	Model 4
Without prosthesis	1 (ref.)	1 (ref.)	1 (ref.)	1 (ref.)
Fixed prosthesis	1.392 (1.244,1.557)	1.379 (1.232,1.543)	1.373 (1.223,1.541)	1.363 (1.213,1.532)
Partial denture	2.655 (2.208,3.193)	2.547 (2.114,3.069)	2.263 (1.873,2.734)	2.275 (1.879,2.753)
Complete denture	3.142 (2.452,4.026)	2.95 (2.298,3.788)	2.502 (1.946,3.217)	2.483 (1.929,3.197)
*p* for trend	<0.0001	<0.0001	<0.0001	<0.0001

Model 1 was adjusted for age and gender; Model 2 was adjusted for age, gender, smoking, drinking, hypertension, diabetes, and BMI; Model 3 was adjusted for age, gender, smoking, drinking, hypertension, diabetes, BMI, education, and income; Model 4 was adjusted for age, gender, smoking, drinking, hypertension, diabetes, BMI, education, income, and daily frequency of toothbrushing.

## Data Availability

The data that support the findings of this study are available from the Korean National Health and Nutrition Examination Survey at https://knhanes.cdc.go.kr/knhanes/eng/index.do.
